# ABCC5, ERCC2, XPA and XRCC1 transcript abundance levels correlate with cisplatin chemoresistance in non-small cell lung cancer cell lines

**DOI:** 10.1186/1476-4598-4-18

**Published:** 2005-05-09

**Authors:** David A Weaver, Erin L Crawford, Kristy A Warner, Fadel Elkhairi, Sadik A Khuder, James C Willey

**Affiliations:** 1 Department of Medicine, Medical College of Ohio, 3055 Arlington Ave., Toledo, OH 43699, USA

## Abstract

**Background:**

Although 40–50% of non-small cell lung cancer (NSCLC) tumors respond to cisplatin chemotherapy, there currently is no way to prospectively identify potential responders. The purpose of this study was to determine whether transcript abundance (TA) levels of twelve selected DNA repair or multi-drug resistance genes (*LIG1*, *ERCC2*, *ERCC3*, *DDIT3*, *ABCC1*, *ABCC4*, *ABCC5*, *ABCC10*, *GTF2H2*, *XPA*, *XPC *and *XRCC1*) were associated with cisplatin chemoresistance and could therefore contribute to the development of a predictive marker. Standardized RT (StaRT)-PCR, was employed to assess these genes in a set of NSCLC cell lines with a previously published range of sensitivity to cisplatin. Data were obtained in the form of target gene molecules relative to 10^6 ^β-actin (*ACTB*) molecules. To cancel the effect of *ACTB *variation among the different cell lines individual gene expression values were incorporated into ratios of one gene to another. Each two-gene ratio was compared as a single variable to chemoresistance for each of eight NSCLC cell lines using multiple regression. In an effort to validate these results, six additional lines then were evaluated.

**Results:**

Following validation, single variable models best correlated with chemoresistance (p < 0.001), were *ERCC2/XPC*, *ABCC5/GTF2H2*, *ERCC2/GTF2H2*, *XPA/XPC *and *XRCC1/XPC*. All single variable models were examined hierarchically to achieve two variable models. The two variable model with the highest correlation was (*ABCC5/GTF2H2*, *ERCC2/GTF2H2*) with an R^2 ^value of 0.96 (p < 0.001).

**Conclusion:**

These results provide markers suitable for assessment of small fine needle aspirate biopsies in an effort to prospectively identify cisplatin resistant tumors.

## Background

Non-small cell lung cancer (NSCLC) is the most common type of bronchogenic carcinoma. Although chemotherapeutic regimens with greater efficacy continue to be developed, the best regimens presently give an overall response rate of only 30–50%. Lack of response is attributable to resistance that is present *de novo *or develops in response to treatment. If the resistance to drugs could be surmounted or if the most effective drug candidates for treatment could be better determined, the impact in terms of survival would be substantial. Because mechanisms of chemoresistance likely involve multiple gene products, we hypothesize that patterns of individual gene expression and/or indices comprising the expression values of multiple genes will provide more effective markers of chemoresistant NSCLC tumors than values of individual genes.

Currently, cisplatin and carboplatin are among the most widely used cytotoxic anticancer drugs. However, resistance to these drugs through *de novo *or induced mechanisms undermines their curative potential [1]. Recently, understanding regarding potential modes of chemoresistance to platinum compounds has been obtained through studies correlating cytotoxicity with nucleotide excision-repair (NER) [2-7] or drug uptake/efflux [7-13]. In this study, we investigated whether *de novo *gene expression differences are correlated with a predisposition of NSCLC tumors to chemoresistance.

Current advances in technology, including microarrays and quantitative RT-PCR methods, enable classification of cancer types on the basis of TA levels rather than histomorphology [14,15]. For example, these techniques enable the discovery of predictive markers based on TA profiles. Microarray screening analysis currently is being investigated to predict chemotherapeutic sensitivity based on TA profiles [16-18]. An advantage of microarray analysis is that thousands of genes may be simultaneously evaluated. However, it is generally recognized that, due to lack of standardization, relatively low sensitivity and relatively poor lower thresholds of detection, microarray assessments need to be confirmed with follow-up quantitative methods. StaRT-PCR is a method that enables rapid, sensitive, reproducible, standardized, quantitative measurements for many genes simultaneously [19,49,50].

Briefly, in StaRT-PCR, the TA level of each gene is made relative to an internal standard (IS) within a standardized mixture of internal standards (SMIS). Known concentrations of these mixtures are combined with cDNA samples in a master mixture for PCR amplification. This enables quantitative measurement of gene expression while controlling for inter-sample, inter-experimental and loading differences. With StaRT-PCR, due to the presence of the SMIS, the measurements are quantitative and quality-controlled when measured either kinetically or at endpoint [51,52]. In other words, measurement of each TA value relative to a known quantity of internal standard controls for variation in amplification efficiency in early, log-linear, and plateau phases of PCR [53].

In an initial survey, StaRT-PCR was used to measure expression of 35 genes involved in DNA repair, multi-drug resistance, cell cycling and apoptosis in two cell lines previously reported to be the least (H460) and most (H1435) chemoresistant among 20 NSCLC cell lines [20]. It was determined that genes involved in DNA repair (*ERCC2*, *XRCC1*) and drug influx/efflux (*ABCC5*) were associated with chemoresistance. The number of genes from each of these two categories was expanded to include additional representative genes associated with generalized DNA damage recognition and repair (*DDIT3*), associated specifically with NER (*LIG1*, *ERCC3*, *GTF2H2*, *XPA*, *XPC*), or associated with drug transport (*ABCC1*, *ABCC4*, *ABCC10*). Expression of these twelve genes was measured in eight NSCLC cell lines with variable cisplatin resistance [20]. StaRT-PCR data were obtained using *ACTB *as a reference gene. Thus, data were reported in the form of mRNA molecules/10^6 ^*ACTB *molecules. These data then were combined into interactive transcript abundance indices (ITAI) by placing one or more genes directly associated with the phenotype on the numerator and one or more genes negatively associated with the phenotype on the denominator [19,21]. It is reasonable to expect that optimal predictors of phenotypes are more likely to be discovered among ITAI than among expression levels of individual genes. This has been demonstrated for certain cancer-related phenotypes [19,21-23]. A further advantage of ITAI is that they control for previously observed variation in the reference gene value (in this case, *ACTB*) from one cell line to another [19,21]. When a single gene in the numerator is divided by another single gene in the denominator, the reference value mathematically cancels out. The ITAI values were compared to cisplatin chemoresistance among the eight NSCLC cell lines with variable resistance. Results then were validated in an additional six NSCLC cell lines.

## Results

### Reproducibility

Among the gene expression measurements for which three or more replicate values were obtained, the mean coefficient of variation was 38.5% (see Additional file 1). This is similar to the reproducibility observed in other gene expression studies using the StaRT-PCR method [19,22]. Recently, through implementation of robotic liquid handlers, automation software, and standard operating procedures in the NCI funded (CA95806) Standardized Expression Measurement (SEM) Center, variation among replicates has been reduced to a CV of less than 10% [50].

### Individual gene expression measurements and chemoresistance

The results of the direct comparison of individual gene expression mean values versus cisplatin chemoresistance are presented in Table 1. All StaRT-PCR data values were in the form of molecules/10^6 ^*ACTB *molecules (see Additional file 1). For 8/12 genes assessed, the correlation was significant (p < 0.05).

**Table 1:** Correlation between each of twelve putative chemoresistance transcript abundance values and chemoresistance among NSCLC cell lines. Eight of the 12 selected multi-drug resistance and DNA repair genes were significantly correlated with chemoresistance among the first group of 8 NSCLC cell lines evaluated (Group 1). In order to validate these results, an additional six lines were evaluated (Group 2).

	**Group 1^*a*^**		**Groups 1 and 2^*b*^**	
		
**Gene**	**R^2^**	**p value**	**R^2^**	**p value**
				
ABCC5	0.93	0.0001	0.85	0.0001
ERCC2	0.85	0.0012	0.82	0.0001
XPA	0.81	0.0023	0.75	0.0001
LIG1	0.78	0.0034	NA^*c*^	NA
DDIT3	0.68	0.0120	NA	NA
ABCC1	0.64	0.0164	NA	NA
XRCC1	0.56	0.0318	0.57	0.0019
ERCC3	0.56	0.0319	NA	NA
ABCC4	0.37	0.1081	NA	NA
ABCC10	0.06	0.5641	NA	NA
GTF2H2	0.02	0.7424	0.04	0.5183
XPC	0.00	0.9851	0.02	0.6654

^*a *^Initial 8 NSCLC cell lines analyzed. ^*b *^All 14 NSCLC cell lines analyzed. ^*c*^Not Assessed.

### Establishment of inter-active transcript abundance indices

ITAI were established as balanced ratios comprising every possible combination with one gene divided by the TA value of another gene for data obtained from each of the initial eight NSCLC cell lines (Group 1). Each TA value was calculated as molecules/10^6 ^*ACTB *molecules. Thus, in these ITAI, the effect of the reference gene, *ACTB*, is cancelled. For example: *ERCC2 *molecules/10^6 ^*ACTB *molecules ÷ *XPC *molecules/10^6 ^*ACTB *molecules = *ERCC2 *molecules/*XPC *molecules. Bivariate analysis of each two-gene ratio versus corresponding cisplatin IC_50 _chemoresistance value was conducted among the eight cell lines (see Additional file 2). There were 12 genes assessed and 11 sets of ratios for each gene as the numerator resulting in 132 ratios. The data from bivariate analyses then were ranked in descending order such that the ratio set listed first was that for which the mean value for correlation with chemoresistance was highest, and the ratio set listed last was that for which the mean r value for correlation with chemoresistance was lowest. Thus, the ratio set with *ERCC2 *in the numerator is listed first because the mean r value for the ratios between *ERCC2 *and each of the other eleven genes was the most positive among the twelve genes evaluated. In contrast, the ratio set with *XPC *in the numerator is listed last because the ratios between *XPC *and each of the other 11 genes had the most negative correlation with chemoresistance.

### Modelling of gene expression with chemoresistance

The ratios *ERCC2/XPC*, *ABCC5/GTF2H2*, *ERCC2/XRCC1*, *ERCC2/GTF2H2*, *XPA/XPC*, *XRCC1/XPC*, and *ABCC5/XPC *were the best (i.e. those single variable models with highest R^2 ^identified in the initial eight NSCLC cell lines by simple linear regression (see Additional file 2). The effect of adding a second variable into the model was then assessed. The best two variable model was (*ABCC5/GTF2H2*, *ERCC2/GTF2H2*) with an R^2 ^value of 0.96.

### Validation of Models

We tested our single and two variable models in an additional six NSCLC cell lines (Table 2). In statistical analysis of the combined data for all 14 NSCLC cell lines, the p value improved or stayed the same for three of the single variable models (*ERCC2/XPC*, *ABCC5/GTF2H2*, *XRCC1/XPC*), as well as the two variable model. The decline in p value for *ERCC2/GTF2H2 *and *XPA/XPC *was not significant. In contrast, ERCC2/XRCC1 was no longer significantly associated with chemoresistance, and the p value declined substantially for *ABCC5/XPC*.

**Table 2:** Bivariate correlation between two-transcript abundance ratios and chemoresistance in a validation set of NSCLC cell lines. The ratios best correlated with chemoresistance from Additional file 2 were evaluated in an additional six lines. The effect of adding a second variable into the model was assessed. ERCC2/XPC was no longer correlated with chemoresistance and ABCC5/XPC had substantially lower p value. The other single variable models and the two variable model were validated.

**Model**	**Group 1^*a*^**		**Groups 1 and 2^*b*^**	
		
**Single variable**	**Model r^2^**	**p value**	**Model r^2^**	**p value**
				
ERCC2/XPC	0.91	0.0002	0.69	0.0002
ABCC5/GTF2H2	0.91	0.0002	0.88	0.0001
ERCC2/XRCC1	0.90	0.0003	NS^*c*^	
ERCC2/GTF2H2	0.90	0.0004	0.63	0.0007
XPA/XPC	0.89	0.0004	0.62	0.0008
XRCC1/XPC	0.88	0.0005	0.75	0.0001
ABCC5/XPC	0.88	0.0006	0.29	0.0461
				
**Two variable**	**Model r^2^**	**p value**	**Model r^2^**	**p value**
				
ABCC5/GTF2H2 and ERCC2/GTF2H2	0.96	0.0003	0.91	0.0001

^*a *^Initial 8 NSCLC cell lines analyzed. ^*b *^All 14 NSCLC cell lines analyzed. ^*c*^not significant (p > 0.05).

## Discussion

The results obtained by measuring gene expression with StaRT-PCR, incorporating values for individual genes into ITAI, and correlating ITAI with chemoresistance led us to propose several models as potential predictors of cisplatin chemoresistance in cultured NSCLC cells. These models comprise genes that have been associated with cisplatin chemoresistance in previous studies including *ABCC5 *[13], and *XPA *[4,24].

Experimental results suggest that increased expression of *ABCC5*, also known as *MRP5*, is associated with exposure to platinum drugs in lung cancer *in vivo *and/or the chronic stress response to xenobiotics [13]. Thus, increased resistance to platinum drugs with increased *ABCC5 *levels may be due to glutathione *S*-platinum complex efflux. Increased efflux of platinum drugs could result in lower levels of drug available to form damaging DNA-platinum drug adducts.

*XPA *and *ERCC2 *are components of the nucleotide excision repair (NER) mechanism, which generally is recognized as the major repair response to DNA damage induced by chemotherapeutic agents such as cisplatin [1,3,7]. In NER, *XPA *is the main DNA lesion recognition protein [25], is the key element in assembly of the NER complex by recruiting several other proteins to the lesion site [26] and *XPA *levels are rate-limiting for NER [4,27]. Enhanced NER gene expression is a major cause of resistance to cisplatin and other DNA-damaging chemotherapeutic agents [3,28] and over expression of the *XPA *gene component of NER has been associated with resistance to cisplatin in human ovarian cancer [4,24]. *ERCC2 *specifically is a component of the transcription factor IIH (TFIIH) that consists of seven polypeptides [29,30] and in its entirety is a repair factor [31-33]. In NER, *ERCC2 *(or *XPD*) is essential for TFIIH helicase activity [34] and it has been demonstrated more recently that *ERCC2 *interacts specifically with *GTF2H2 *(or *p44*) and that this interaction results in the stimulation of the 5' to 3' helicase activity [35]. In at least some other tissues, *ERCC1 *is associated with cisplatin resistance, while *ERCC2 *is not [36,37]. Thus, our data support the importance of excision repair in cisplatin resistance, but suggest that there is inter-tissue variation in the excision repair genes that are responsible for *de novo *cisplatin resistance.

*XRCC1 *has long been recognized as a key component of the base excision repair (BER) pathway, acting as a "scaffold" for the coordination of other BER proteins at the sites of base damage during repair [38-40]. It has been shown that polymorphisms in *XRCC1*, while in themselves are not associated with increased risk of lung cancer, have shown an increased risk of lung cancer in a supermultiplicative manner when associated with polymorphisms in another component of BER, poly (ADP-ribose) polymerase family, member 1 transfersase (*PARP1*) [41]. *XRCC1 *has also recently been proposed as a component of an alternative nonhomologous end-joining route of DNA double-stranded breaks (DSBs), that complements the predominant repair pathway of DNA-dependent protein kinase (DNA-PK) and X-ray repair complementing defective repair in Chinese hamster cells 4 (*XRCC4*)-DNA ligase IV complex [42]. Although the NER pathway is the major repair mechanism for cisplatin-DNA adducts, our data supports the proposal of overlapping repair pathways involved in alternative repair of cisplatin adducts, such as the BER pathway. *XRCC1 *may also be involved in the repair of other types of DNA damage caused by cisplatin including DSBs.

Selection of a stable reference for the amount of sample loaded for each gene expression measurement is important to ensure measurement accuracy and reproducibility. With microarray analysis, because thousands of genes are assessed simultaneously, an index of all genes measured provides a stable reference for the amount of sample loaded from one microarray to another. In quantitative RT-PCR studies, typically, a single non-regulated gene is used as a loading reference, such as *ACTB*, *GAPD*, cyclophilin or ribosomal RNA. However, all of these genes have been reported to vary among multiple samples. One way to assess inter-sample variation in reference gene expression among multiple samples is to compare variation between two reference genes. In our experience, *ACTB *and *GAPD *vary 50-fold relative to each other among bronchial epithelial cells (BEC) and even more between BEC and other cell types [19,44]. In situations where limited numbers of genes are measured (< 200), an index of all genes for the normalization of data is not sufficiently stable. In order to eliminate the effect of unknown variation in the reference gene expression among samples, we analyzed balanced ratios of one gene expression value obtained by StaRT-PCR to another. These balanced ratios did not represent actual cellular concentration changes of the individual genes comprising the ratio, but related the expression of one gene to another and could be used for comparison with phenotypic determinants such as chemoresistance. In this study, ITAI analysis (Table 2) confirmed most of the results obtained by analysis of individual gene expression values relative to chemoresistance (Table 1). This suggests that variation in *ACTB *among this group of cDNA samples was not significant. However, in our experience inter-sample variation in *ACTB *expression is greater among primary samples. Thus, we will continue to use ITAI to remove doubt regarding potential effect of variation in reference gene expression whenever possible.

As is presented in Table 2, by evaluating an empirically derived set of balanced ratios (ITAI) derived from expression values for all of the genes measured, it is possible to establish a hierarchy regarding the strength of association between a set of genes and a phenotype.

## Conclusion

In summary, the association of *ERCC2*, *ABCC5*, *XPA*, and *XRCC1 *with chemoresistance was established through a sequential process involving a) screening genes representing many different functional classes, b) evaluating an expanded group of genes represented by those that were positively associated in the first round, c) identification of outliers (see Additional file 2), d) model building and e) validation (Table 2). Although only two of the 35 genes assessed in the first round were correlated with chemoresistance, 8/12 of the selected DNA repair and MDR genes were correlated. The models established in this study demonstrate the importance of evaluating the interaction among multiple genes representing multiple pathways involved in cisplatin chemoresistance. These models will be tested through a blinded study of gene expression levels of the identified potential markers in samples consisting of fine needle aspirate (FNA) biopsies from patients with various treatment outcomes.

## Methods

### Cell culture

Non-small cell lung cancer (NSCLC) cell lines with varying levels of cisplatin chemoresistance, H460, H1155, H23, H838, H1334, H1437, H1355, H1435, H358, H322, H441, H522, H226 and H647, were obtained from the American Type Culture Collection (Rockville, MD). The previously reported [20] cisplatin IC_50 _concentration for each line is provided in Table 3. All cells were incubated in RPMI-1640 medium (Biofluids, Inc., Rockville, MD) containing 10% fetal bovine serum (FBS) and 1 mM glutamine at 37°C in the presence of 5% CO_2_. Proliferative, subconfluent cultures were obtained for RNA extractions and subsequent analyses.

**Table 3:** Cisplatin chemoresistance in 14 non-small cell cancer cell lines

	**NSCLC cell line**	**Cisplatin IC_50 _(μM)**
**Group 1^*b*^**	NCI-H460	0.52 ± 0.04
	NCI-H1155	0.90 ± 0.20
	NCI-H23	2.09 ± 0.32
	NCI-H838	3.86 ± 0.31
	NCI-H1334	5.15 ± 0.47
	NCI-H1437	5.90 ± 1.61
	NCI-H1355	6.74 ± 1.29
	NCI-H1435	22.86 ± 2.36

**Group 2^*c*^**	NCI-H358	1.16 ± 0.24
	NCI-H322	2.85 ± 0.22
	NCI-H441	3.38 ± 0.99
	NCI-H522	3.53 ± 0.56
	NCI-H226	5.05 ± 0.95
	NCI-H647	7.27 ± 1.14

^*a *^Previously published results by Chun-Ming Tsai, et.al. [20]

^*b *^Initial set of 8 NSCLC cell lines evaluated.

^*c *^Additional set of 6 NSCLC cell lines evaluated.

### Reagents

10X PCR buffer for the Rapidcycler (500 mM Tris, pH 8.3; 2.5 mg/μl BSA; 30 mM MgCl_2_) was obtained from Idaho Technology, Inc. (Idaho Falls, ID). Taq polymerase (5 U/μl), oligo dT primers, RNasin (25 U/μl) and dNTPs were obtained from Promega (Madison, WI). M-MLV reverse transcriptase (200 U/μl) and 5X first strand buffer (250 mM Tris-HCl, pH 8.3; 375 mM KCl; 15 mM MgCl_2_; 50 mM DTT) were obtained from Gibco BRL (Gaithersburg, MD). DNA 7500 Assay kits containing dye, matrix and standards were obtained from Agilent Technologies, Inc. (Palo Alto, CA). All other chemicals and reagents were molecular biology grade.

### RNA extraction and reverse transcription

Total RNA was isolated from cell cultures by a TriReagent protocol (Molecular Research Center, Inc., Cincinnati, OH) [43]. Following extraction, approximately 1 μg of total RNA for each cell line was reverse-transcribed using M-MLV reverse-transcriptase and an oligo dT primer as previously described [44].

### Quantitative standardized RT (StaRT)-PCR

Gene expression was determined using previously published quantitative StaRT-PCR protocols [19,44-50]. Briefly, a master mixture containing buffer, MgCl_2_, dNTPs, sample cDNA, Taq polymerase and SMIS was prepared and 9 μl aliquots dispensed into 0.6 ml microfuge tubes containing 1 μl of gene-specific primers. A SMIS comprises gene-specific IS's for each gene at defined concentrations relative to one another. The mixture includes IS's for reference (or housekeeping genes) to control for cDNA loading and to simplify normalization of all gene data. All primers used for PCR and those used in the construction of the CTs, are listed in Additional file 3. PCR reactions mixtures were subjected to 35 cycles of PCR with 5 seconds of denaturation at 94°C, 10 seconds of annealing at 58°C and 15 seconds of elongation at 72°C in a Rapidcycler (Idaho Technology, Inc.). PCR products were electrophoretically separated and quantified in an Agilent 2100 Bioanalyzer (Agilent Technologies, Inc.) with the DNA 7500 Assay kit. The area under the curve (as calculated by Agilent software) for each native template (NT) and IS peak was used in all calculations. Representative electropherograms of each gene assessed are presented in Additional file 4. The NT/IS ratio for a reference gene, *ACTB*, and the NT/IS ratios for each target gene were calculated. The initial number of NT molecules for each gene then could be determined from these ratios because the initial number of IS molecules added into the PCR reaction was known. To normalize measurements and control for sample-to-sample variation and inter-experimental loading, the calculated number of target gene molecules was divided by the calculated number of *ACTB *molecules. A size correction was employed to correct for fluorescence intensity differences affecting the measured area under the curve [19,48].

### Statistical analyses

Ratios of one gene to another, from each of the initial eight NSCLC cell lines, were subjected to multiple regression analysis using SAS 6.12 (SAS Institute Inc., Cary, NC) to determine the combination of genes that best predict cisplatin resistance. Each ratio was compared separately to chemoresistance and ratios with significant correlation to resistance (R^2 ^≥ 0.88, p < 0.001) then were examined hierarchically to achieve two variable models based on the highest R^2 ^values. Following assessment of an additional 6 cell lines, results for all 14 NSCLC cell lines were combined and also subjected to analysis as described.

## Abbreviations

*LIG1 *– DNA ligase I, ATP-independent; *ERCC2 *– excision repair cross-complementing rodent repair deficiency (ERCC), complementation group 2; *ERCC3 *– ERCC, complementation group 3; *DDIT3 *– DNA-damage-inducible transcript 3; *ABCC1 *– ATP-binding cassette, subfamily C (ABCC), member 1; *ABCC4 *– ABCC, member 4; *ABCC5 *– ABCC, member 5; *ABCC10 *– ABCC, member 10; *GTF2H2 *– general transcription factor IIH, polypeptide 2; *XPA *– xeroderma pigmentosum (XP), complementation group A; *XPC *– XP, complementation group C and *XRCC1 *– X-ray repair complementing defective repair in Chinese hamster cells 1.

## Authors' contributions

DAW participated in study design, cell culture, transcript abundance analysis and data interpretation and drafted the manuscript. ELC participated in data interpretation and manuscript preparation. KAW participated in cell culture and study design. FE conducted gene expression experiments. SAK conducted statistical analyses and participated in study design and data interpretation. JCW conceived of the study, participated in study design and data interpretation, and critically reviewed the manuscript.

## Competing interests

DAW, ELC, KAW and JCW each have significant equity interest in Gene Express, Inc. which produces and markets StaRT-PCR reagents used in these studies.

## Supplementary Material

Additional File 1 StaRT-PCR Average Transcript Abundance Data Click here for file

Additional File 2 Bivariate correlation between two-transcript abundance ratios and chemoresistance in the eight NSCLC lines of Group 1 Click here for file

Additional File 3 Primers used for StaRT-PCR analysis Click here for file

Additional File 4 Representative electropherograms for detminations of transcript abundance Click here for file
