# Supplementary material for: ABCC5, ERCC2, XPA and XRCC1 transcript abundance levels correlate with cisplatin chemoresistance in non-small cell lung cancer cell lines
Source: Mol Cancer. 2005 May 9;4:18. doi: 10.1186/1476-4598-4-18 (PMC1156938; doi:10.1186/1476-4598-4-18)
Supplement: Additional File 1 — StaRT-PCR Average Transcript Abundance Data [file 1476-4598-4-18-S1.pdf]

Supplemental Table 1

NSCLC cell line	Group number[a]	cisIC50[b]	LIG1[c]	ERCC2	ERCC3	DDIT3	ABCC1	ABCC4	ABCC5	ABCC10	GTF2H2	XPA	XPC	XRCC1
**H460**	1	0.52	3.53E+02	2.54E+02	3.07E+02	1.00E+02	7.37E+02	3.50E+02	5.90E+01	1.80E+01	1.02E+03	3.10E+02	5.25E+02	1.88E+03
**H1155**	1	0.9	5.90E+02	5.81E+02	1.27E+03	1.26E+03	3.84E+02	3.30E+02	9.00E+01	4.30E+01	1.09E+03	1.65E+03	2.33E+03	4.04E+03
**H23**	1	2.09	7.74E+02	8.29E+02	1.81E+03	1.55E+03	3.13E+02	1.87E+03	1.39E+02	4.56E+02	4.42E+03	1.18E+03	1.44E+03	8.27E+03
**H838**	1	3.86	1.16E+03	8.69E+03	1.81E+03	5.79E+02	6.21E+03	1.35E+03	4.01E+02	5.12E+02	4.18E+03	2.23E+03	5.36E+03	3.14E+04
**H1334**	1	5.15	4.50E+02	1.28E+03	7.66E+02	6.17E+02	5.48E+02	6.05E+02	9.90E+01	1.17E+02	1.33E+03	5.07E+02	1.18E+03	6.53E+03
**H1437**	1	5.9	2.56E+02	6.13E+02	3.72E+02	4.59E+02	8.48E+02	4.10E+01	2.64E+02	2.00E+02	1.74E+03	5.84E+02	7.30E+02	2.84E+03
**H1355**	1	6.74	9.03E+02	6.52E+02	1.01E+03	3.75E+02	1.48E+03	1.10E+03	2.67E+02	8.30E+01	1.04E+03	1.34E+03	1.37E+03	4.57E+03
**H1435**	1	22.86	2.59E+03	3.01E+04	3.37E+03	3.76E+03	9.24E+03	2.24E+03	1.39E+03	3.31E+02	2.84E+03	6.77E+03	1.93E+03	4.19E+04
**H358**	2	1.16		1.32E+03					6.80E+01		1.02E+03	1.34E+03	1.03E+03	4.02E+03
**H322**	2	2.85		4.02E+03					4.50E+01		6.63E+02	6.64E+02	5.51E+02	3.87E+03
**H441**	2	3.38		1.19E+03					1.57E+02		1.14E+03	6.11E+02	7.28E+02	2.96E+03
**H522**	2	3.53		7.12E+02					4.03E+02		2.46E+03	4.83E+02	4.54E+02	2.41E+03
**H226**	2	5.05		8.85E+02					1.04E+02		1.10E+03	5.97E+02	3.52E+03	2.46E+03
**H647**	2	7.27		4.64E+03					1.92E+02		8.84E+02	1.06E+03	2.71E+03	1.04E+04

[a] Group 1 is the initial set of 8 NSCLC cell lines evaluated. Group 2 is the additional set fo 6 NSCLC cell lines evaluated.

[b] Previously published results by Chun-Ming Tsai, et.al. [20]

[c] All mean values resulting from triplicate experimental determinations are reported as the number of molecules of the gene listed per 10^6 molecules of ACTB.
