# Supplementary material for: ABCC5, ERCC2, XPA and XRCC1 transcript abundance levels correlate with cisplatin chemoresistance in non-small cell lung cancer cell lines
Source: Mol Cancer. 2005 May 9;4:18. doi: 10.1186/1476-4598-4-18 (PMC1156938; doi:10.1186/1476-4598-4-18)
Supplement: Additional File 2 — Bivariate correlation between two-transcript abundance ratios and chemoresistance in the eight NSCLC lines of Group 1 [file 1476-4598-4-18-S2.doc]

## Additional file 2. Bivariate correlation between two-transcript abundance ratios and chemoresistance in the eight NSCLC lines of Group 1

Data from bivariate analyses were ranked in descending order. They were ranked first by ratio sets generated for each gene as the numerator relative to the other 11 genes, and second by r value for each individual ratio.
