# Supplementary material for: ABCC5, ERCC2, XPA and XRCC1 transcript abundance levels correlate with cisplatin chemoresistance in non-small cell lung cancer cell lines
Source: Mol Cancer. 2005 May 9;4:18. doi: 10.1186/1476-4598-4-18 (PMC1156938; doi:10.1186/1476-4598-4-18)
Supplement: Additional File 3 — Primers used for StaRT-PCR analysis [file 1476-4598-4-18-S3.doc]

**Additional file 3. – Primers used for StaRT-PCR analysis**
